# Quality of life and outcomes in heart failure patients with ejection fractions in different ranges

**DOI:** 10.1371/journal.pone.0218983

**Published:** 2019-06-27

**Authors:** Xin Chen, Yanguo Xin, Wenyu Hu, Yinan Zhao, Zixin Zhang, Yinpin Zhou

**Affiliations:** 1 Department of Cardiology, The First Affiliated Hospital, China Medical University, Shenyang, China; 2 Department of Cardiology, Fuling Central Hospital, Chongqing, China; 3 Department of Cardiology, West China Hospital of Sichuan University, Chengdu, China; Providence VA Medical Center, UNITED STATES

## Abstract

**Aims:**

Guidelines divide patients with heart failure (HF) into 3 distinct groups based on left ventricular ejection fraction (LVEF) We used the Minnesota Living with Heart Failure Questionnaire (MLHFQ) to quantify the health-related quality of life in patients with HF.

**Methods:**

Patients were stratified into three cohorts: preserved LVEF (>50%), mid-range LVEF (40–49%) and reduced LVEF (<40%). The MLHFQ scores were evaluated using one-way ANOVA, and differences were observed among the groups. The association of New York Heart Association (NYHA) class with the physical scores was analyzed by Spearman’s correlation analysis. The predictive utility of the total MLHFQ scores was assessed with Kaplan-Meier curves for death and HF-related hospitalization. The Cox proportional hazards model was used to identify the risk factors for prognosis. Internal reliability was assessed with Cronbach’s α.

**Results:**

There were significant differences in the total MLHFQ scores and the MLHFQ subscale scores among the three groups (p<0.05). MLHFQ domains demonstrated high internal consistency among the three groups (Cronbach’s α = 0.92, 0.96 and 0.93). The MLHFQ physical subscale scores were significantly associated with NYHA class in HFrEF (r = 0.59, p<0.001) and HFmrEF patients (r = 0.537, p<0.001). The survival analysis indicated that there was a significant difference among the three groups regarding high MLHFQ scores (p = 0.038). In the groups with low MLHFQ scores, the HFmrEF group exhibited significantly increased rates of death and HF-related hospitalization compared with the HFpEF group (p = 0.035).

**Conclusions:**

The features and clinical outcomes varied among heart failure patients with different EF values. The MLHFQ appears to be a valid and reliable measurement of health status and offers excellent prognostic ability.

## Introduction

The number of patients with heart failure is increasing due to the aging population and the therapeutic advancements that improve the survival of patients with heart diseases. HF has been a major cause of mortality and morbidity[1]. LVEF is used to measure systolic function and has been the central determinant prognostic factor in heart failure[2]. Approximately one-third to half of all patients with heart failure have preserved ejection fraction[3]. The features, triggers, prognosis, and response to therapy of patients with preserved EF are different from those with reduced EF. Some studies demonstrated that patients with preserved EF have a lower mortality rate and a lower hospitalization rate than those without[2, 4, 5]. However, other clinical trials[6, 7] have reported opposite conclusions, reporting that patients with preserved EF may have worse prognosis in terms of hospitalization and mortality compared with those with reduced EF. Meanwhile, several studies have assessed the features and clinical outcomes of patients with borderline EF[8]. The latest guidelines published by the European Society of Cardiology (ESC) recommended separating patients with heart failure into three distinct groups depending on their LVEF: preserved LVEF (≥50%), mid-range LVEF (40–49%), and reduced LVEF (<40%)[9]; however, the comparison of clinical outcomes among patients with different EF ranges was limited.

The health-related quality of life (HRQoL) of patients with HF is an important outcome as it reflects the impact of HF on their daily lives. Heart failure is an end-stage of various cardiovascular diseases, and therefore, being able to qualifying patients’ physical and emotional statuses is important and must be reliable for physicians to evaluate the effect of therapy. Additionally, improving HRQoL is an important goal in heart failure treatment. There is less information on the comparisons of HRQoL in these three groups of heart failure patients. In recent decades, various specific HRQoL questionnaires for patients with HF have been regarded as important assessment tools for how heart failure impacts their symptoms, function and quality of life[10–13]. The MLHFQ is one of the most widely used health-related quality of life questionnaires for patients with heart failure[14, 15].

In this retrospective study, we evaluated health-related quality of life with the MLHFQ among three groups of patients that were stratified by LVEF (preserved LVEF, mid-range LVEF and reduced LVEF), providing an opportunity to study the HRQoL of heart failure patients in these three populations.

## Methods

From November 2014 to August 2015, a total of 875 hospitalized patients who were diagnosed with heart failure at The First Affiliated Hospital of China Medical University were enrolled in this study, regardless of ejection fraction and etiology. All patients involved provided informed consent, and our work was approved by the Ethics Committee of China Medical University, and was carried out in accordance with the Code of Ethics of the World Medical Association. HF was diagnosed based on standard guideline criteria[9]; namely, relevant symptoms plus objective evidence of cardiac dysfunction. Key exclusion criteria included patients who died during hospitalization, had uncontrolled hypertension, had constrictive pericarditis, and had any organic mental or psychiatric disorders that might hinder the completion of the questionnaire.

Of the 875 patients enrolled, 12 were lost to follow-up, and 22 refused to complete the questionnaire. A total of 841 patients were included in this study. Detailed information regarding the patients was collected, including etiology, clinical stage, medications, device therapies, comorbidities and New York Heart Association (NYHA) functional class; a physician-assigned assessment of the patients’ symptoms was also recorded for each patient. All 841 enrolled patients were separated into three groups according to their value of EF, including preserved EF (LVEF≥50%) (n = 251), mid-range LVEF (40–49%) (n = 267), and reduced LVEF (<40%) (n = 323) (S1 Fig). Patients were assessed for disease-specific health status according to the MLHFQ; the MLHFQ is a self-administered disease-specific questionnaire for patients with HF, comprising 21 items representing different degrees of impact of HF on HRQoL, from 0 (none) to 5 (very much). It provides a total score (range 0–105), and scores for two dimensions, physical limitations (questions 2–7 and 12–12, range 0–40) and emotional limitations (questions 17–21, range 0–25). These questions cover symptoms and signs that are relevant to HF, and a higher score indicates worse quality of life. The questionnaire was performed to evaluate the stability of patients’ health status by the time of discharge. During the 12-month follow-up period, all included patients would complete the questionnaire again. The primary endpoints including all-cause mortality and HF-related hospitalization were examined during follow-up. All clinical outcomes were collected by telephone or during a clinic visit six and 12 months after discharge from the hospital.

### Statistical methods

Categorical data are presented as frequencies (percentages), and continuous data are presented as the mean values ± standard deviations (SDs). The chi-square test or Fisher’s exact test was used to compare categorical variables. The differences between the three groups were tested with ANOVA. Spearman’s rank-order correlations were used to compare the relationships between the MLHFQ score and the NYHA functional class across all three groups. To evaluate the prognostic ability of the MLHFQ, Kaplan-Meier curves with a log-rank p values were used to calculate cumulative events based on the MLHFQ scores: the first quartile, the second quartile, the third quartile and the fourth quartile. All variables underwent univariate analysis, and the factors with p<0.20 in the univariate analysis and those that had a significant impact on the prognosis according to clinical experience were included in a stepwise Cox proportional hazard model analysis to estimate the potential factors involved in the interaction analysis and the analysis of the interaction of HF types with the MLHFQ scores. The Hazard Ratio (HRs) and 95% confidence intervals (CIs) were calculated. Statistical interactions were tested by multiple regression models, and subgroup survival analysis was carried out by a Kaplan-Meier analysis. All p-values were 2-sided, and p<0.05 was considered statistically significant.

### Internal reliability of the MLHFQ

Cronbach’s alpha was used to determine the internal consistency of the MLHFQ domains among patients in the three subgroups, separately. Cronbach’s alpha evaluates the internal consistency of the items within a domain. Values ranged from 0 to 1, with larger values providing greater consistency[16]. A value ≧0.70 was considered satisfactory for internal consistency.

## Results

Table 1 contains detailed patient characteristics and comparisons among the three groups. The following statistically significant differences were observed: the HFrEF group was slightly older (66.92 vs 64.77 vs 63.53, p = 0.028); heart rates in HFrEF and HFpEF groups were slightly higher (72.77 vs 70.42 vs 71.35, p = 0.001); the HFrEF and HFpEF groups had lower eGFR (78.4 vs 84.1 vs 80.1, p = 0.014).

**Table 1:** Baseline characteristics of each heart failure group.

	HFrEF (n = 323)	HFmrEF (n = 267)	HFpEF (n = 251)	P value
Age(years)	66.92±10.8	64.77±12.64	63.53±13.26	0.028
Male (%)	187(58.01%)	153(57.40%)	155(61.76%)	0.784
Heart rate, bpm	72.77±13.4	70.42±8.15	71.35±10.63	0.001
**Comorbidities (%)**				
Hypertension	197(61.07%)	173(64.81%)	138(54.91%)	0.334
Diabetes	150(46.56%)	121(45.37%)	108(43.14%)	0.872
Previous MI	128(39.69%)	111(41.67%)	103(41.18%)	0.949
Stroke/TIA	111(34.35%)	72(26.85%)	64(25.49%)	0.267
alcohol	84(25.95%)	91(34.26%)	84(33.33%)	0.308
Smoking	175(54.20%)	129(48.15%)	120(48.04%)	0.548
Previous HF	217(67.1%)	178(66.7%)	164(65.3%)	0.895
previous AF	61(18.8%)	45(16.8%)	43(17.1%)	0.780
**Laboratory values**				
Serum sodium, mmol/L	133.5±4.15	134.6±3.88	134.3±4.81	0.541
Creatinine, umol/L	93.6±37.44	85.4±49.98	83.1±26.69	0.121
eGFR, ml/min	78.4±33.78	84.1±44.11	80.1±27.59	0.014
Hemoglobin, g/l	109±15.5	112.7±16.6	112.3±14.6	0.178
BNP, pg/ml	1845(1657–2033)	1441(1240–1642)	798(633–947)	0.061
**NYHA functional class**				0.007
II	77(23.6%)	67(25%)	81(32.4%)	
III	197(61.1%)	188(70.4%)	160(63.7%)	
IV	49(15.3%)	12(4.6%)	10(3.9%)	
LVESD	61.6±3.84	58.53±4.33	54.1±4.33	0.132
ACEI/ARB	244(75.57%)	203(75.93%)	219(87,25%)	0.056
Beta-blocker	232(71.76%)	200(75%)	212(84.31%)	0.073
Loop diuretics	286(88.55%)	235(87.96%)	197(78.43%)	0.062
Spironolactone	202(62.60%)	165(60.19%)	182(72.55%)	0.137
digoxin	89(27.48%)	69(25.93%)	81(32.35%)	0.559

MI: myocardial infarction; TIA: transient ischemic attack; eGFR: estimated glomerular filtration rate; BNP: brain natriuretic peptide; NYHA: New York Heart Association; EF: ejection fraction; LVESD: left ventricular end systolic diameter; ACEI: angiotensin converting enzyme inhibitors; ARB: angiotensin Ⅱ receptor blocker

### Internal consistency of the MLHFQ

In patients with HFrEF, the degree of internal consistency evaluated with Cronbach’s alpha in each MLHFQ domain was large (α>0.80); Cronbach’s α coefficients in the MLHFQ ranged from a low minimum of 0.86 (physical subscale) to a maximum of 0.92 (total score). This pattern was also observed among patients with HFpEF and patients with HFmrEF (Table 2).

**Table 2:** Internal consistency of the MLHFQ domains.

	Total patients	HFrEF	HFmrEF	HFpEF
	(n = 841)	(n = 323)	(n = 267)	(n = 251)
**Total score**	0.83	0.92	0.96	0.93
**Physical score**	0.80	0.86	0.93	0.91
**Emotional score**	0.75	0.88	0.91	0.89

MLHFQ: Minnesota living with heart failure questionnaire; HFrEF: heart failure with reduced ejection fraction; HFmrEF: heart failure with mid-range ejection fraction; HFpEF: heart failure with preserved ejection fraction

### Criterion validity of the MLHFQ

We compared the trend of the physical MLHFQ scores based on NYHA class. As shown in Table 3 and S2 Fig, the MLHFQ physical scores were correlated with NYHA class in HFrEF and HFmrEF patients (r = 0.59 and 0.537, respectively; p<0.001), and the physical scores were not correlated with the NYHA class in HFpEF patients (p = 0.552).

**Table 3:** Mean MLHFQ physical score by NYHA class.

	Total patients (n = 841)	HFrEF (n = 323)	HFmrEF (n = 267)	HFpEF (n = 251)
**NYHA II**	11.23(2.52)	14(2.01)	10.5(1.95)	8.46(1.68)
**NYHA III**	16.65(2.78)	21.44(2.67)	18.57(2.12)	14.47(2.31)
**NYHA IV**	25.39(3.03)	27(2.95)	25.25(2.48)	23.5(2.71)
**P value**	0.302	<0.001	<0.001	0.522

MLHFQ: Minnesota living with heart failure questionnaire; NYHA: New York Heart Association; HFrEF: heart failure with reduced ejection fraction; HFmrEF: heart failure with mid-range ejection fraction; HFpEF: heart failure with preserved ejection fraction

### Predictive validity of the MLHFQ

**Fig 1:**
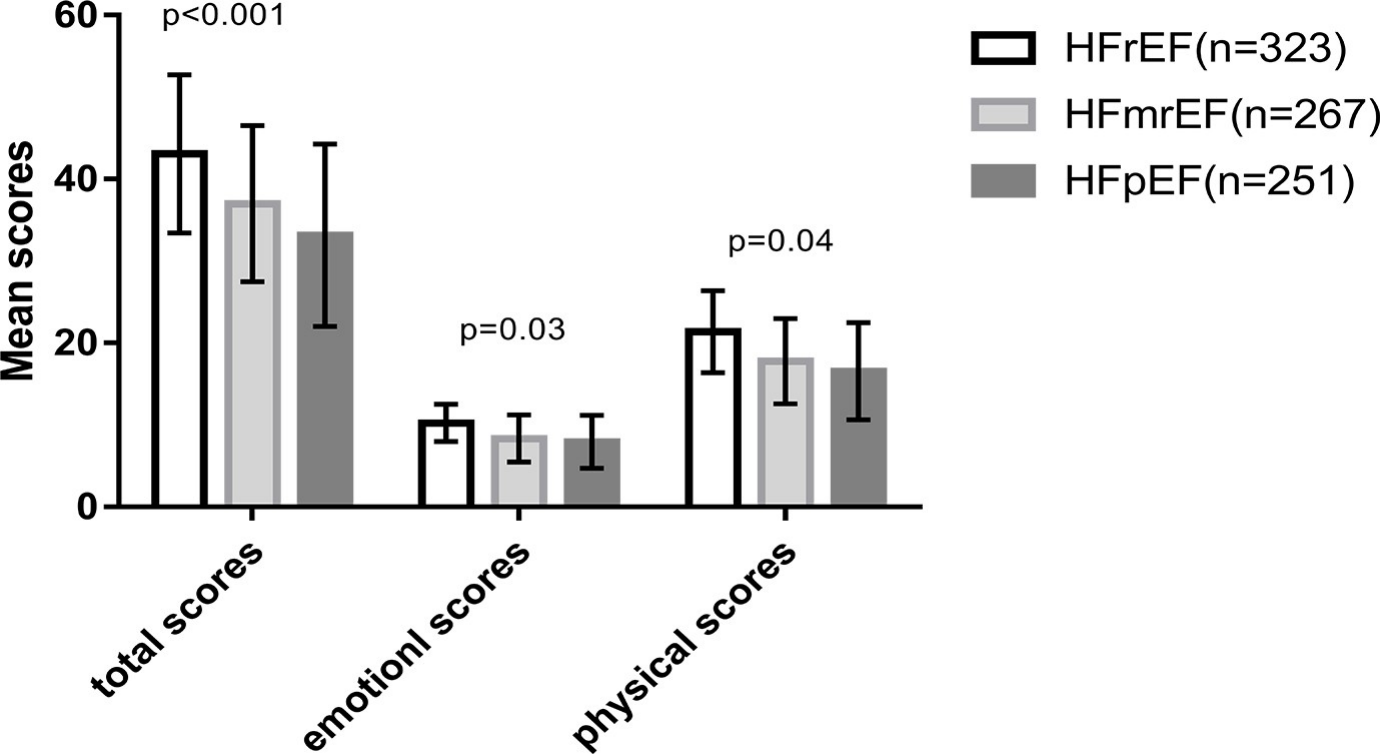
Comparison of MLHFQ scores among three groups with different ejection fraction range.

The MLHFQ total score and subscales demonstrated statistically significant differences among the three groups. There was a significant difference in the total MLHFQ score in all three groups (43.1 vs 36.9 vs 33.2, p<0.001). Patients with HFpEF and HFmrEF presented lower scores on the MLHFQ subscales than those with HFrEF (Fig 1).

**Fig 2:**
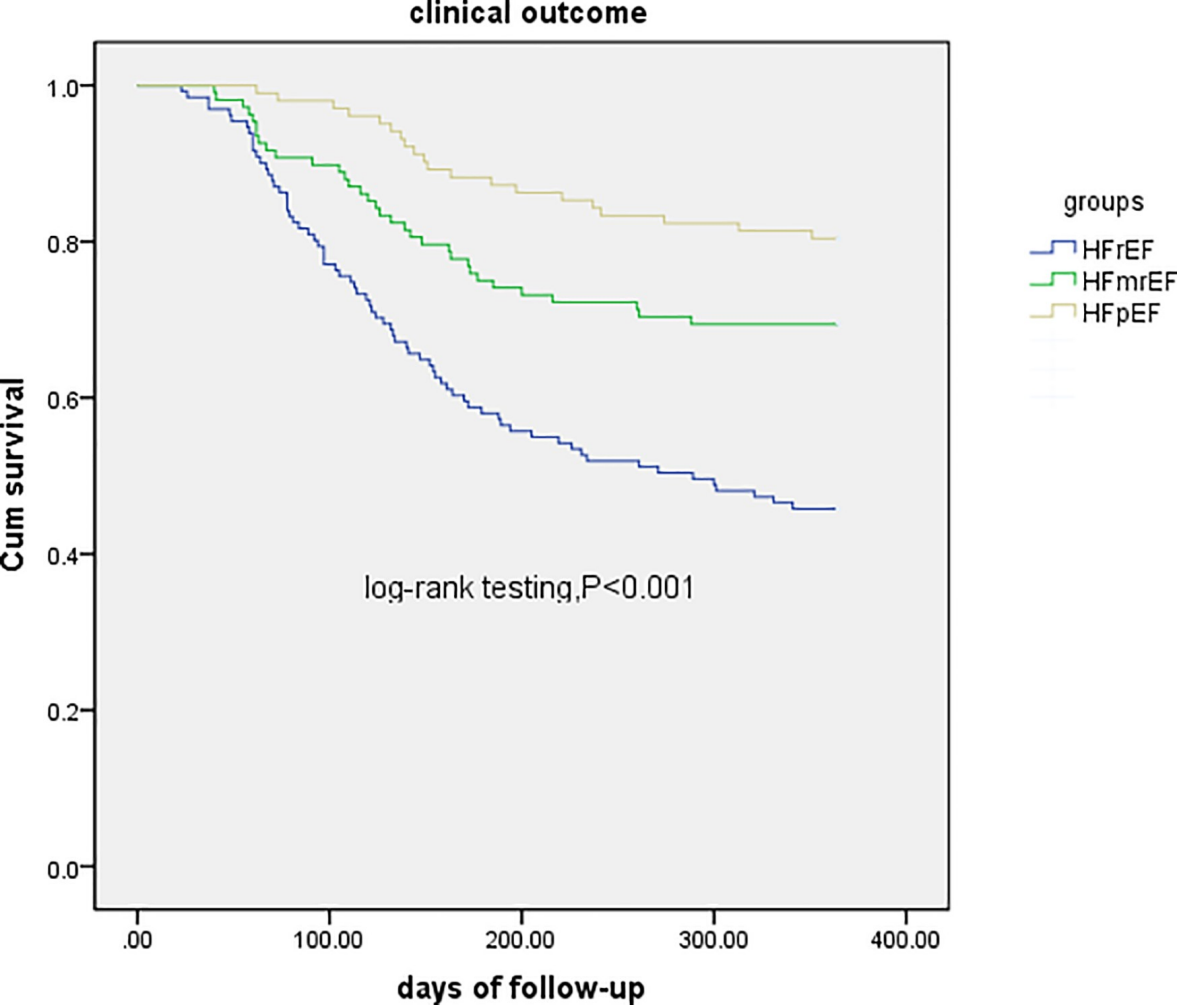
Kaplan-Meier cumulative survival at 1 years’ follow-up.

At the end of the follow-up period, there were a total of 301 outcomes: 165 events in the HFrEF group (54.8%), 76 events in the HFmrEF group (25.2%), and 60 events in the HFpEF group (19.9%). Kaplan-Meier analysis showed that the incidence-free prognosis was significantly better in the HFpEF and HFmrEF groups (log-rank test, p<0.001) (Fig 2).

**Fig 3:**
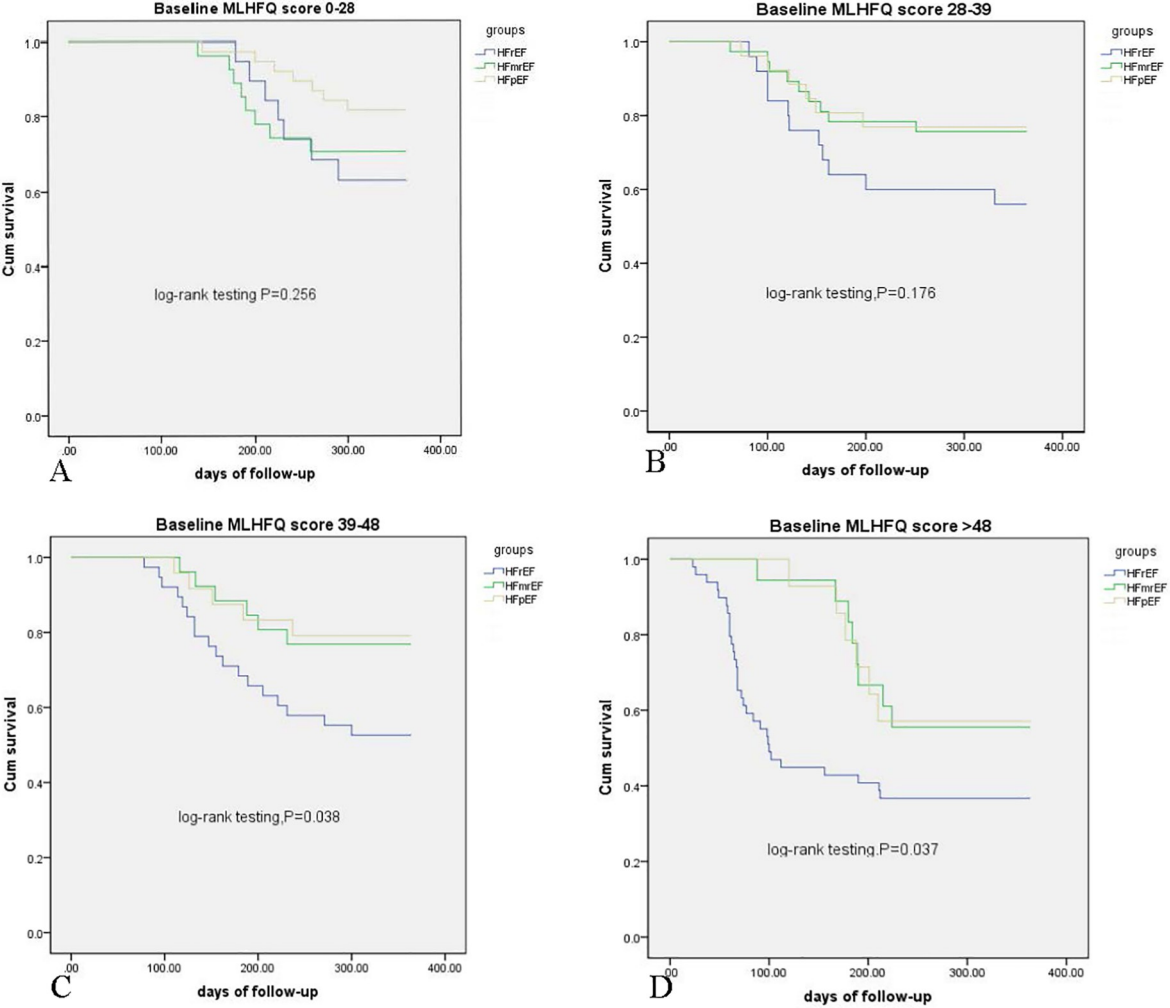
Kaplan-Meier survival analysis by baseline MLHFQ score tercile. (A) Kaplan-Meier survival analysis of the first quartile (score 0–28). (B) Kaplan-Meier survival analysis of the second quartile (score 28–39). (C) Kaplan-Meier survival analysis of the third quartile (score 39–48). (D) Kaplan-Meier survival analysis of the forth quartile (score >48).

We divided the total MLHFQ scores of all patients by quartiles and compared the outcome rates among the three groups at different MLHFQ score levels. Outcome rates at 1 year within the first quartile did not differ significantly among the three groups (log-rank test, p = 0.256) (Fig 3A), but the following pairwise comparison with the first quartile MLHFQ scores between the HFmrEF and HFpEF groups showed that the HFpEF group had a lower rate of outcomes (log-rank test, p = 0.035). Outcome rates at 1 year within the second quartile also did not significantly differ among the three groups (log-rank test, p = 0.176) (Fig 3B). However, in patients with the third and fourth quartile MLHFQ scores, patients with HFpEF and HFmrEF had significantly lower outcome rates at 1 year (log-rank test, p = 0.038 and p = 0.037, respectively) (Fig 3C and 3D).

**Fig 4:**
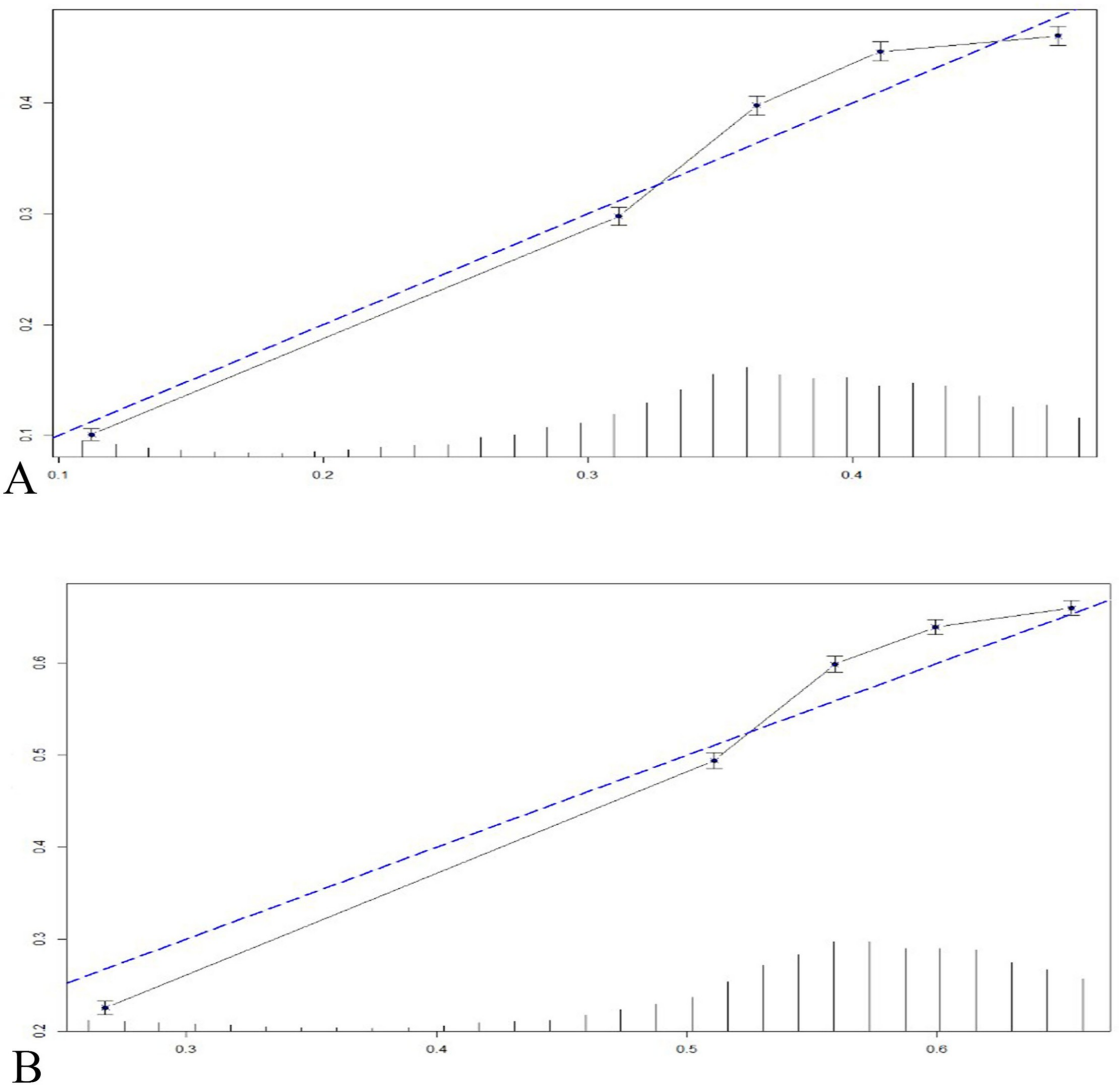
Kaplan-Meier survival analysis of the subgroup of clinical factors.

In the following analysis, we employed an interaction effect to determine the relationship between different clinical factors and different ejection fraction groups. We first identified the potential clinical factors associated with endpoints using a Cox multivariate analysis, and the related factors are shown in Table 4. Five factors including renal function (eGFR), previous MI, heart rate, loop diuretics and beta blocker usage were associated with outcomes. The stratified analyses revealed that participants with eGFR<90ml/min/1.73 m^2^ showed a better prognosis in the HFpEF and HFmrEF groups compared with those in the HFrEF group (HFpEF: HR: 0.602, 95% CI: 0.322–0.901; HFmrEF: HR: 0.480, 95%CI: 0.317–0.883, p<0.001). Patients with previous MI showed similar results (HFpEF: HR: 0.409, 95% CI: 0.215–0.866; HFmrEF: HR: 0.362, 95% CI: 0.201–0.994, p = 0.015). In addition, patients receiving loop diuretics in the HFpEF and HFmrEF groups had lower incidence rates of endpoints. Interestingly, patients with heart rate>70bpm in the HFmrEF group showed a lower incidence of endpoints compared with the HFrEF (HR:0.356, 95% CI: 0.184–0.643, p<0.001). A Kaplan-Meier analysis in the subgroups were consistent with the above results (Fig 4). In the following analysis of the interaction between HF types and MLHFQ score (Table 5), we found that the type of heart failure had an important impact on the total MLHFQ score and on the physical subscale but not on the emotional subscale. Additionally, we found that with increasing scores, the significance was much more obvious among different types of HF.

**Table 4:** Association between clinical factors and endpoints.

subgroup	Hazard Ratio(95%CI) HFpEF vs HFrEF	Hazard Ratio(95%CI) HFmrEF vs HFrEF	P	P for interaction
**Age**				0.158
>70 years old	0.512(0.32,1.082)	0.682(0.277,0.874)	0.032	
<70 years old	0.661(0.371,1.121)	0.874(0.681,1.351)	0.511	
**Sex**				0.221
Male	0.493(0.314,1.021)	1.131(0.579,1.417)	0.216	
Female	0.514(0.348,0.921)	0.374(0.219,0.827)	0.024	
**eGFR**				<0.001
≥90ml/min/1.73m^2^	0.548(0.311,1.170)	0.755(0.421,1.084)	0.117	
**<**90ml/min/1.73m^2^	0.602(0.322,0.901)	0.480(0.317,0.883)	0.021	
**DM**				0.32
No	0.487(0.318,1.271)	0.541(0.355,1.159)	0.21	
Yes	0.733(0.289,1.017)	0.742(0.474,1.221)	0.40	
**Previous MI**				0.015
No	0.512(0.361,1.084)	1.320(0.758,3.430)	0.310	
Yes	0.409(0.215,0.866)	0.362(0.201,0.994)	<0.001	
**Digoxin**				0.831
No	0.641(0.338,1.214)	1.213(0.648,2.187)	0.873	
Yes	0.566(0.289,0.763)	0.301(0.274,0.845)	0.041	
**Heart rate**				0.020
≤70 bpm	0.389(0.224,0.748)	1089(0.748,1.899)	0.356	
>70 bpm	0.746(0.431,1.875)	0.356(0.184,0.643)	<0.001	
**Loop diuretics**				<0.001
No	0.745(0.374,1.321)	0.848(0.626,2.412)	0.306	
Yes	0.886(0.541,0.927)	0.411(0.286,0.875)	0.021	
**ACEI/ARB**				0.512
No	0.665(0.416,1.221)	0.859(0.674,2.186)	0.768	
Yes	0.401(0.189,0.715)	0.454(0.203,0.741)	0.030	
**β-blocker**				0.064
No	1.032(0.899,2.84)	0.894(0.766,1.457)	0.115	
Yes	0.264(0184,0.421)	0.441(0.311,0.726)	<0.001	

eGFR: evaluated glomerular filtration rate;DM:diabetes mellitus; MI: myocardial infarction, ACEI: angiotensin converting enzyme inhibitors, ARB: angiotensin Ⅱ receptor blocker

**Table 5:** Association between HF types and MLHFQ score.

subgroup	Hazard Ratio(95%CI) HFpEF vs HFrEF	Hazard Ratio(95%CI) HFmrEF vs HFrEF	P	P for interaction
**Total scores**				<0.001
0–26	1.051(0.873–1.332)	0.981(0.739–1.432)	0.103	
26–40	0.893(0.768–1.041)	0.979(0.881–1.112)	0.121	
40–54	0.773(0.658–0.946)	0.445(0.387–0.963)	0.024	
>54	0.536(0.239–0.694)	0.401(0.282–0.628)	<0.001	
**Physical scores**				0.047
0–13	1.032(0.672–1.340)	1.178(0.821–1.663)	0.098	
13–19	0.918(0.712–1.025)	0.524(0.319–0.884)	0.069	
19–25	0.789(0.607–0.994)	0.763(0.662–0.802)	0.038	
>25	0.434(0.308–0.769)	0.776(0.623–0.946)	<0.001	
**Emotional score**				0.083
0–6	0.867(0.492–1.163)	0.926(0.685–1.303)	0.231	
6–13	0.749(0.338–1.092)	0.603(0.442–1.150)	0.089	
13–18	0.285(0.194–0.726)	0.640(0.558–1.344)	0.061	
>18	0.464(0.235–0.986)	0.771(0.654–0.921)	0.033	

MLHFQ: Minnesota living with heart failure questionnaire; CI: confidence interval; HFpEF: heart failure with preserved ejection fraction; HFmrEF: heart failure with mid-range ejection fraction; HFrEF: heart failure with reduced ejection fraction.

## Discussion

This present study enrolled patients diagnosed with heart failure at the First Affiliated Hospital of China Medical University; all enrolled patients completed the MLHFQ. This study provided an opportunity to evaluate the HRQoL in patients with heart failure with preserved, mid-range and reduced ejection fraction.

We investigated the emotional and physical scores in all three groups, and we found similar internal reliability in the HFrEF, HFmrEF and HFpEF patients. We also noted that higher MLHFQ scores were associated with worse event-free survival at one year. Moreover, our analyses suggested that the MLHFQ predicted clinical outcomes in patients across the EF spectrum.

Among patients with chronic heart failure, nearly half had normal or near-normal left ventricular ejection fraction. Heart failure with mid-range ejection fraction (40–49%) is receiving more attention. Prior studies have investigated the features, triggers, prognosis and response to therapy in HFmrEF patients[2, 8, 17]. Improving HRQoL is an important goal in heart failure treatment, and many of the HRQoL studies focusing on patients with HFrEF and HFpEF[17, 18] have shown that improving HRQoL is an important treatment objective in HF patients. However, there are limited studies that have evaluated HRQoL among patients with reduced, mid-range and preserved ejection fraction. This study aimed to evaluate the HRQoL of patients with MLHFQ scores among patients with ejection fraction values in different ranges. Previous researches also reported different questionnaires in patients with heart failure[19, 20]. Kansas City Cardiomyopathy Questionnaire (KCCQ) is another widely used questionnaire of HRQoL, Susan et al reported that KCCQ was a valid and reliable measure for HFpEF, and Yashashwi et al offered similar evidence in HFpEF and HFrEF patients. Neither these two articles enrolled patients with HFmrEF. In our study, we reached similar conclusions in HFpEF and HFrEF by MLHFQ, but we also included patients with HFmrEF.

The MLHFQ and the NYHA are two functional classification methods for patients with heart failure. In this study, we evaluated the associations between these two criteria. In patients with HFrEF and HFmrEF, there were many correlations between physical scores and NYHA class. However, there were few correlations in patients with HFpEF. This may be due to the conditions of the patients with HFpEF or due to our data limitations; therefore, further studies will be required to identify the correlation between the MLHFQ and the NYHA in patients with HFpEF.

The goal of the present study was to assess the utility of the MLHFQ in evaluating status and progression in heart failure patients with different ejection fractions. Many trials have focused on evaluating HRQoL in patients with heart failure[19, 21]. Comparing patients with HFpEF and patients with HFmrEF revealed that there was only a significant difference between the groups when the total MLHFQ score was less than 28, and there was no difference in survival when the total score was greater than 28. This may indirectly provide evidence for the idea that heart failure with mid-range ejection fraction is a distinct clinical entity, that can stimulate research into underlying characteristics, pathophysiology and treatment of this group of patients. However, for patients with HFrEF, higher scores are also an important factor with independent prognostic implications for survival and morbidity.

### Limitations

First, in this study, only the MLHFQ was introduced, and there were no additional data on the functional capacity of patients. We tried to assess their physical function by 6-minute walk test, but the data were not statistically analyzed. Second, HRQoL, was only measured with one instrument, and thus, the determinants of HRQoL need to be validated using a different tool. Additional concerns include the time of follow-up and the number of patients enrolled. The follow-up period was 1 year, which is relatively short compared with other studies on HRQoL as a predictor of long-term mortality that have used follow-up periods of 5–7 years. Finally, previous study[22] reported that nearly 70% of patients with HFpEF had recovered from a previously low EF, which is termed heart failure with recovered ejection fraction (HFrecEF). In our study, we divided patients into different groups according to their EF value in stable condition in hospital, but there were no data during the follow-up period.

## Conclusions

HFrEF, HFmrEF and HFpEF patients had different prognoses according to the MLHFQ scores, and the MLHFQ is a reliable instrument for the measurement of health status and quality of life in patients with HF. Additional studies are needed to verify our findings.

## Supporting information

S1 Fig Flow chart of study. (TIF)

S2 Fig Line chart of the relationship between MLHFQ and HF subgroups. (TIF)
