# Supplementary figures and images for: Quality of life and outcomes in heart failure patients with ejection fractions in different ranges
Source: PLoS One. 2019 Jun 27;14(6):e0218983. doi: 10.1371/journal.pone.0218983 (PMC6597164; doi:10.1371/journal.pone.0218983)

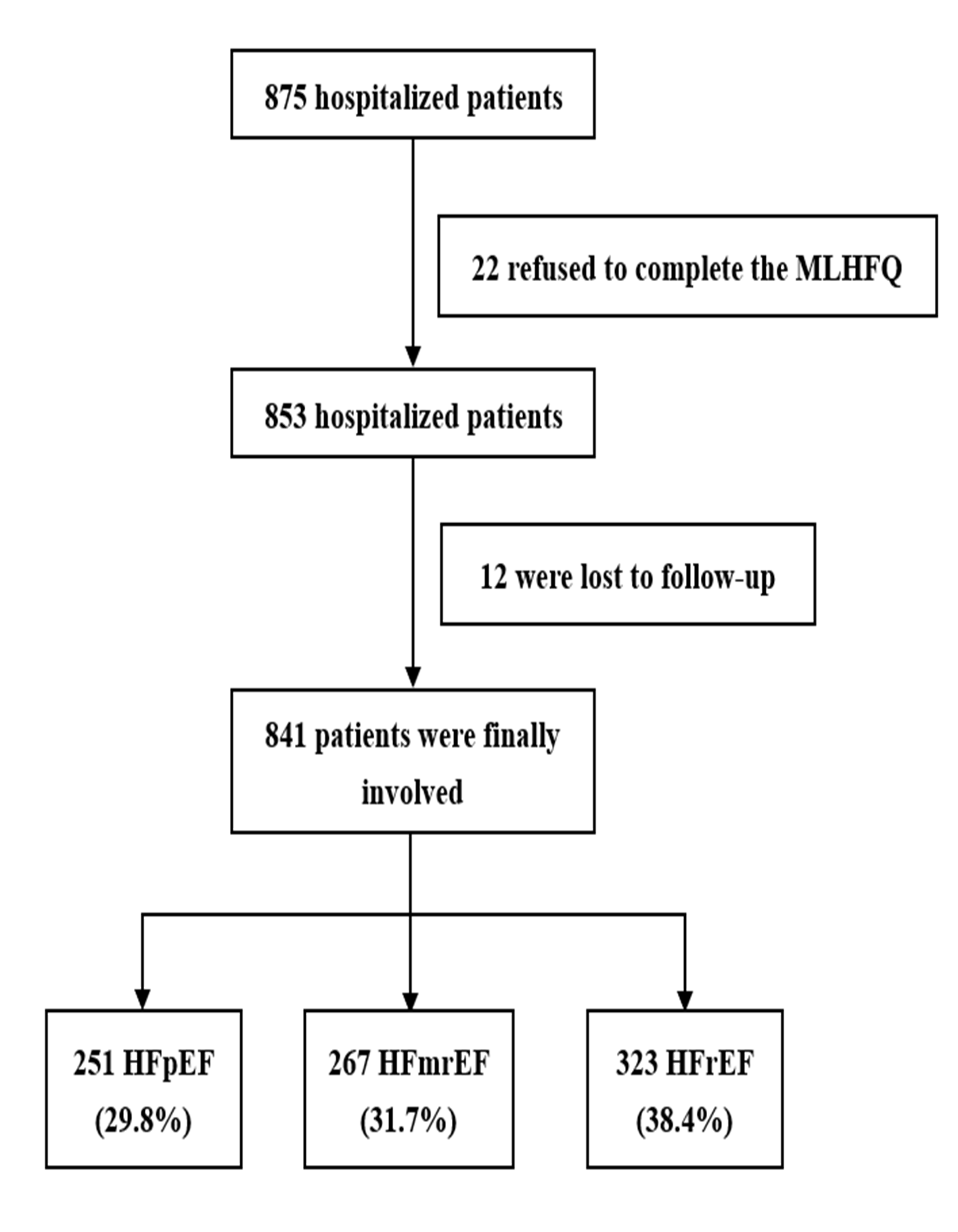

Supplement: S1 Fig — (TIF) [file pone.0218983.s001.tif]

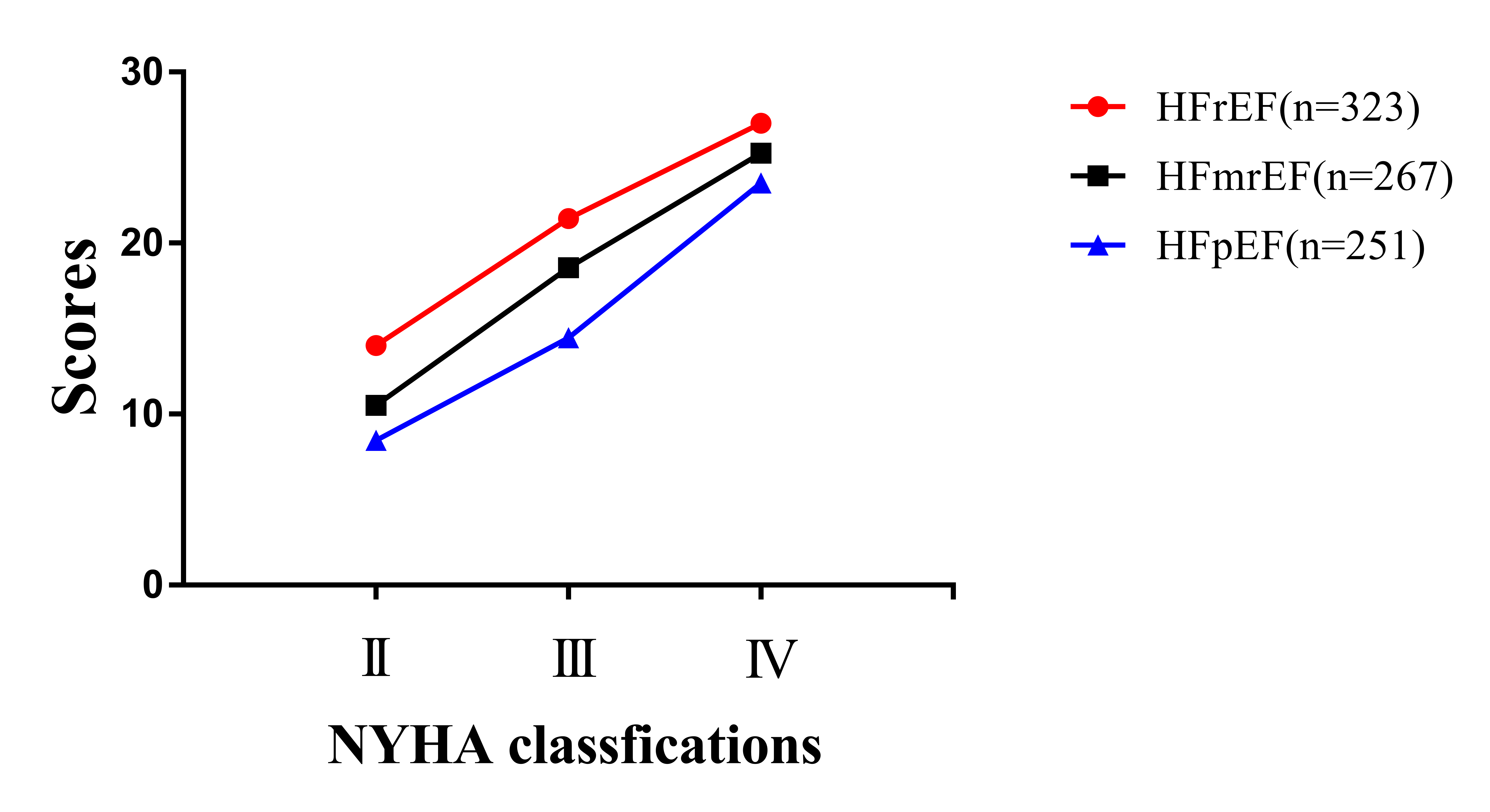

Supplement: S2 Fig — (TIF) [file pone.0218983.s002.tif]
